# Triethyl­ammonium 4-nitro­benzene­sulfonate

**DOI:** 10.1107/S1600536810021379

**Published:** 2010-06-16

**Authors:** Mohammad T. M. Al-Dajani, Hassan H. Abdallah, Nornisah Mohamed, Ching Kheng Quah, Hoong-Kun Fun

**Affiliations:** a School of Pharmaceutical Sciences, Universiti Sains Malaysia, 11800 USM, Penang, Malaysia; b School of Chemical Sciences, Universiti Sains Malaysia, 11800 USM, Penang, Malaysia; c X-ray Crystallography Unit, School of Physics, Universiti Sains Malaysia, 11800 USM, Penang, Malaysia

## Abstract

In the anion of the title molecular salt, C_6_H_16_N^+^·C_6_H_4_O_5_S^−^, the nitro group is twisted slightly from the benzene ring, making a dihedral angle of 3.16 (10)°. In the crystal structure, the cations and anions are linked into a two-dimensional network parallel to the *ab* plane by C—H⋯O and N—H⋯O hydrogen bonds.

## Related literature

For general background to and the synthesis of the title compound, see: Dann & Davies (1929 ▶); D’Souza *et al.* (2008 ▶); Hunig *et al.* (1965 ▶); Kim *et al.* (1999 ▶). For the stability of the temperature controller used for the data collection, see: Cosier & Glazer (1986 ▶). For a related structure, see: Quah *et al.* (2008 ▶).
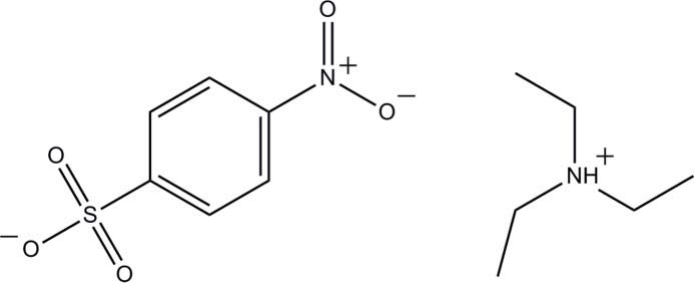


## Experimental

### 

#### Crystal data

C_6_H_16_N^+^·C_6_H_4_NO_5_S^−^
*M* *_r_* = 304.36
Orthorhombic, 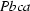

*a* = 7.8015 (14) Å
*b* = 12.669 (2) Å
*c* = 29.910 (6) Å
*V* = 2956.3 (9) Å^3^
*Z* = 8
Mo *K*α radiation
μ = 0.24 mm^−1^
*T* = 100 K
0.22 × 0.18 × 0.14 mm

#### Data collection

Bruker SMART APEXII DUO CCD area-detector diffractometer
Absorption correction: multi-scan (*SADABS*; Bruker, 2009 ▶) *T*_min_ = 0.950, *T*_max_ = 0.967
21787 measured reflections
5605 independent reflections
3985 reflections with *I* > 2σ(*I*)
*R*_int_ = 0.064

#### Refinement

*R*[*F* ^2^ > 2σ(*F* ^2^)] = 0.046
*wR*(*F* ^2^) = 0.135
*S* = 1.01
5605 reflections
261 parameters
All H-atom parameters refined
Δρ_max_ = 0.55 e Å^−3^
Δρ_min_ = −0.47 e Å^−3^

### 

Data collection: *APEX2* (Bruker, 2009 ▶); cell refinement: *SAINT* (Bruker, 2009 ▶); data reduction: *SAINT*; program(s) used to solve structure: *SHELXTL* (Sheldrick, 2008 ▶); program(s) used to refine structure: *SHELXTL*; molecular graphics: *SHELXTL*; software used to prepare material for publication: *SHELXTL* and *PLATON* (Spek, 2009 ▶).

## Supplementary Material

Crystal structure: contains datablocks global, I. DOI: 10.1107/S1600536810021379/wn2392sup1.cif

Structure factors: contains datablocks I. DOI: 10.1107/S1600536810021379/wn2392Isup2.hkl

Additional supplementary materials:  crystallographic information; 3D view; checkCIF report

## Figures and Tables

**Table 1:** Hydrogen-bond geometry (Å, °)

*D*—H⋯*A*	*D*—H	H⋯*A*	*D*⋯*A*	*D*—H⋯*A*
N2—H1*N*2⋯O4	0.95 (2)	1.86 (2)	2.7899 (17)	166 (2)
C2—H2*A*⋯O5^i^	0.96 (2)	2.485 (19)	3.1000 (19)	121.7 (14)
C7—H7*B*⋯O4^ii^	1.00 (2)	2.50 (2)	3.4081 (19)	151.3 (16)
C10—H10*B*⋯O3^iii^	0.96 (2)	2.59 (2)	3.461 (2)	151.1 (18)
C12—H12*A*⋯O5^iii^	0.99 (2)	2.60 (2)	3.559 (2)	164.1 (16)

Symmetry codes: (i) 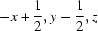; (ii) 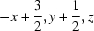; (iii) 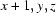.

**Fig. 1.**
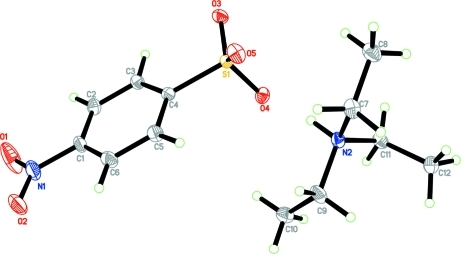
The structures of the two ions of the title compound, showing 50% probability displacement ellipsoids for non-H atoms and the atom-numbering scheme. Hydrogen atoms are shown as spheres of arbitrary radius.

**Fig. 2.**
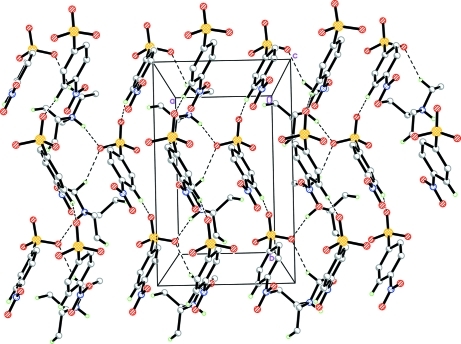
The crystal structure of the title compound viewed along the c axis. H atoms not involved in intermolecular interactions (dashed lines) have been omitted for clarity.

## supplementary crystallographic information

### Comment

Aromatic nitro sulfonyl compounds are very important since they can be used as raw materials for the manufacture of sulfanilamide preparations. *o*-Nitrobenzenesulfonylhydrazide (NBSH) is a very important reagent for the synthesis of allenes from propargylic alcohols. The preparation of NBSH from *o*-nitrobenzenesulfonyl chloride and hydrazine in benzene was described in 1929 by Dann and Davies (Dann & Davies, 1929; D'Souza *et al.*, 2008; Hunig *et al.*, 1965; Kim *et al.*, 1999).

The asymmetric unit (Fig. 1) of the title compound contains one triethylammonium cation and one 4-nitrobenzenesulfonate anion. A proton transfer from the sulfonic acid group of 4-nitrobenzenesulfonic acid to atom N2 of triethylamine resulted in the formation of ions. In the anion, the nitro group is twisted slightly from the attached ring; the dihedral angle between the C1—C6 and O1/O2/N1/C1 planes is 3.16 (10)°. The bond lengths and angles in the 4-nitrobenzenesulfonate anion are within normal ranges and similar to those in a comparable crystal structure (Quah *et al.*, 2008). In the crystal structure, the cations and anions are linked to form a two-dimensional network (Fig. 2) parallel to the *ab*-plane by C—H···O and N—H···O hydrogen bonds (Table 1).

### Experimental

4-Nitrobenzenesulfonyl chloride (0.01 mol, 2.05 g) was dissolved in 25 ml of tetrahydrofuran (THF) in a round-bottomed flask with stirring. Triethylamine (0.01 mol, 0.70 g) was mixed with some THF and added to the flask dropwise with stirring. The reaction mixture was refluxed for 2.5 h and left at room temperature overnight. The needle crystals that were formed were then filtered off, washed with water and dried at 353 K.

### Refinement

All H atoms were located in a difference Fourier map and refined freely [N2—H1N2 = 0.95 (2) Å and C—H = 0.92 (3) - 1.03 (2) Å].

### Crystal data

**Table:**

C_6_H_16_N^+^·C_6_H_4_NO_5_S^−^	*F*(000) = 1296
*M**_r_* = 304.36	*D*_x_ = 1.368 Mg m^−^^3^
Orthorhombic, *P**b**c**a*	Mo *K*α radiation, λ = 0.71073 Å
Hall symbol: -P 2ac 2ab	Cell parameters from 2801 reflections
*a* = 7.8015 (14) Å	θ = 2.7–30.9°
*b* = 12.669 (2) Å	µ = 0.24 mm^−^^1^
*c* = 29.910 (6) Å	*T* = 100 K
*V* = 2956.3 (9) Å^3^	Block, yellow
*Z* = 8	0.22 × 0.18 × 0.14 mm

### Data collection

**Table:**

Bruker SMART APEXII DUO CCD area-detector diffractometer	5605 independent reflections
Radiation source: fine-focus sealed tube	3985 reflections with *I* > 2σ(*I*)
graphite	*R*_int_ = 0.064
φ and ω scans	θ_max_ = 33.2°, θ_min_ = 1.4°
Absorption correction: multi-scan (*SADABS*; Bruker, 2009)	*h* = −8→12
*T*_min_ = 0.950, *T*_max_ = 0.967	*k* = −9→19
21787 measured reflections	*l* = −40→46

### Refinement

**Table:**

Refinement on *F*^2^	Primary atom site location: structure-invariant direct methods
Least-squares matrix: full	Secondary atom site location: difference Fourier map
*R*[*F*^2^ > 2σ(*F*^2^)] = 0.046	Hydrogen site location: inferred from neighbouring sites
*wR*(*F*^2^) = 0.135	All H-atom parameters refined
*S* = 1.01	*w* = 1/[σ^2^(*F*_o_^2^) + (0.0731*P*)^2^] where *P* = (*F*_o_^2^ + 2*F*_c_^2^)/3
5605 reflections	(Δ/σ)_max_ < 0.001
261 parameters	Δρ_max_ = 0.55 e Å^−^^3^
0 restraints	Δρ_min_ = −0.47 e Å^−^^3^

### Special details

**Table:**

Experimental. The crystal was placed in the cold stream of an Oxford Cyrosystems Cobra open-flow nitrogen cryostat (Cosier & Glazer, 1986) operating at 100.0 (1) K.
Geometry. All e.s.d.'s (except the e.s.d. in the dihedral angle between two l.s. planes) are estimated using the full covariance matrix. The cell e.s.d.'s are taken into account individually in the estimation of e.s.d.'s in distances, angles and torsion angles; correlations between e.s.d.'s in cell parameters are only used when they are defined by crystal symmetry. An approximate (isotropic) treatment of cell e.s.d.'s is used for estimating e.s.d.'s involving l.s. planes.
Refinement. Refinement of *F*^2^ against ALL reflections. The weighted *R*-factor *wR* and goodness of fit *S* are based on *F*^2^, conventional *R*-factors *R* are based on *F*, with *F* set to zero for negative *F*^2^. The threshold expression of *F*^2^ > σ(*F*^2^) is used only for calculating *R*-factors(gt) *etc*. and is not relevant to the choice of reflections for refinement. *R*-factors based on *F*^2^ are statistically about twice as large as those based on *F*, and *R*- factors based on ALL data will be even larger.

### Fractional atomic coordinates and isotropic or equivalent isotropic displacement parameters (Å^2^)

**Table:**

	*x*	*y*	*z*	*U*_iso_*/*U*_eq_	
S1	0.38577 (5)	0.17193 (3)	0.095990 (11)	0.01330 (9)	
O1	0.2417 (3)	−0.17343 (11)	0.26000 (4)	0.0524 (5)	
O2	0.37185 (19)	−0.05239 (12)	0.29767 (4)	0.0372 (3)	
O3	0.25347 (14)	0.13583 (9)	0.06573 (3)	0.0195 (2)	
O4	0.55993 (14)	0.14889 (8)	0.07988 (3)	0.0172 (2)	
O5	0.36886 (15)	0.28080 (8)	0.11063 (4)	0.0206 (2)	
N1	0.3145 (2)	−0.08793 (12)	0.26271 (4)	0.0265 (3)	
C1	0.3331 (2)	−0.02431 (12)	0.22174 (5)	0.0184 (3)	
C2	0.2606 (2)	−0.06215 (11)	0.18258 (5)	0.0174 (3)	
C3	0.27633 (19)	−0.00053 (11)	0.14418 (5)	0.0157 (3)	
C4	0.36392 (18)	0.09467 (10)	0.14563 (4)	0.0134 (2)	
C5	0.4376 (2)	0.13060 (12)	0.18557 (5)	0.0187 (3)	
C6	0.4220 (2)	0.07070 (13)	0.22423 (5)	0.0214 (3)	
N2	0.79571 (17)	0.31410 (9)	0.08449 (4)	0.0151 (2)	
C7	0.7094 (2)	0.42076 (11)	0.08501 (5)	0.0192 (3)	
C8	0.6060 (2)	0.44251 (13)	0.04323 (6)	0.0239 (3)	
C9	0.8950 (2)	0.29787 (13)	0.12722 (5)	0.0212 (3)	
C10	0.9187 (2)	0.18278 (14)	0.13903 (6)	0.0242 (3)	
C11	0.9015 (2)	0.29386 (12)	0.04305 (5)	0.0166 (3)	
C12	1.0456 (2)	0.37245 (13)	0.03620 (5)	0.0206 (3)	
H2A	0.195 (3)	−0.1264 (16)	0.1819 (6)	0.023 (5)*	
H3A	0.215 (3)	−0.0242 (14)	0.1159 (6)	0.021 (5)*	
H5A	0.498 (3)	0.2009 (16)	0.1862 (6)	0.022 (5)*	
H6A	0.471 (3)	0.0962 (17)	0.2514 (7)	0.035 (6)*	
H7A	0.641 (3)	0.4184 (16)	0.1120 (7)	0.027 (5)*	
H7B	0.807 (3)	0.4712 (16)	0.0893 (6)	0.020 (5)*	
H8A	0.679 (3)	0.4491 (16)	0.0171 (6)	0.027 (5)*	
H8B	0.517 (3)	0.3859 (17)	0.0374 (7)	0.028 (5)*	
H8C	0.529 (3)	0.5072 (17)	0.0482 (7)	0.028 (5)*	
H9A	1.007 (3)	0.3338 (14)	0.1233 (6)	0.020 (5)*	
H9B	0.830 (3)	0.3364 (16)	0.1497 (7)	0.027 (5)*	
H10A	0.981 (3)	0.1804 (17)	0.1669 (8)	0.039 (6)*	
H10B	0.989 (3)	0.1457 (18)	0.1178 (7)	0.036 (6)*	
H10C	0.817 (4)	0.147 (2)	0.1432 (7)	0.042 (6)*	
H11A	0.825 (3)	0.2928 (14)	0.0187 (6)	0.014 (4)*	
H11B	0.954 (3)	0.2213 (15)	0.0474 (6)	0.016 (4)*	
H12A	1.143 (3)	0.3620 (15)	0.0569 (6)	0.021 (5)*	
H12B	1.008 (3)	0.4425 (17)	0.0365 (6)	0.026 (5)*	
H12C	1.100 (3)	0.3593 (19)	0.0071 (8)	0.036 (6)*	
H1N2	0.702 (3)	0.2662 (15)	0.0832 (6)	0.017 (4)*	

### Atomic displacement parameters (Å^2^)

**Table:**

	*U*^11^	*U*^22^	*U*^33^	*U*^12^	*U*^13^	*U*^23^
S1	0.01496 (15)	0.01173 (14)	0.01322 (16)	0.00071 (12)	−0.00004 (11)	0.00010 (10)
O1	0.0965 (15)	0.0357 (8)	0.0251 (7)	−0.0290 (9)	−0.0008 (8)	0.0093 (5)
O2	0.0421 (8)	0.0529 (9)	0.0166 (6)	−0.0118 (7)	−0.0060 (5)	0.0093 (5)
O3	0.0202 (5)	0.0233 (5)	0.0152 (5)	−0.0025 (4)	−0.0050 (4)	0.0020 (4)
O4	0.0169 (5)	0.0156 (4)	0.0191 (5)	0.0004 (4)	0.0036 (4)	−0.0003 (4)
O5	0.0293 (6)	0.0124 (4)	0.0199 (5)	0.0041 (4)	0.0022 (4)	−0.0009 (4)
N1	0.0325 (8)	0.0300 (7)	0.0170 (6)	−0.0024 (6)	0.0024 (6)	0.0060 (5)
C1	0.0217 (7)	0.0208 (6)	0.0127 (6)	0.0010 (6)	0.0023 (5)	0.0034 (5)
C2	0.0213 (7)	0.0147 (6)	0.0163 (6)	−0.0011 (6)	0.0024 (5)	−0.0002 (5)
C3	0.0182 (6)	0.0154 (6)	0.0135 (6)	0.0005 (5)	0.0006 (5)	−0.0010 (4)
C4	0.0138 (6)	0.0139 (5)	0.0126 (6)	0.0020 (5)	0.0004 (5)	−0.0007 (4)
C5	0.0218 (7)	0.0177 (6)	0.0165 (7)	−0.0038 (6)	−0.0013 (5)	−0.0013 (5)
C6	0.0248 (8)	0.0255 (7)	0.0139 (6)	−0.0031 (6)	−0.0035 (6)	−0.0012 (5)
N2	0.0185 (6)	0.0132 (5)	0.0136 (5)	−0.0021 (5)	0.0002 (4)	0.0006 (4)
C7	0.0242 (7)	0.0130 (6)	0.0203 (7)	0.0007 (6)	0.0035 (6)	−0.0009 (5)
C8	0.0271 (8)	0.0218 (7)	0.0229 (8)	0.0061 (7)	0.0008 (6)	0.0042 (6)
C9	0.0294 (8)	0.0224 (7)	0.0119 (6)	−0.0032 (6)	−0.0020 (6)	0.0014 (5)
C10	0.0215 (8)	0.0275 (8)	0.0234 (8)	0.0041 (7)	−0.0023 (6)	0.0059 (6)
C11	0.0215 (7)	0.0165 (6)	0.0117 (6)	0.0018 (6)	0.0004 (5)	−0.0013 (5)
C12	0.0199 (7)	0.0234 (7)	0.0183 (7)	−0.0008 (6)	0.0025 (6)	0.0018 (5)

### Geometric parameters (Å, °)

**Table:**

S1—O3	1.4468 (11)	N2—H1N2	0.95 (2)
S1—O5	1.4531 (11)	C7—C8	1.513 (2)
S1—O4	1.4709 (11)	C7—H7A	0.97 (2)
S1—C4	1.7865 (14)	C7—H7B	1.00 (2)
O1—N1	1.226 (2)	C8—H8A	0.97 (2)
O2—N1	1.2233 (19)	C8—H8B	1.01 (2)
N1—C1	1.4738 (19)	C8—H8C	1.03 (2)
C1—C2	1.386 (2)	C9—C10	1.512 (2)
C1—C6	1.391 (2)	C9—H9A	0.99 (2)
C2—C3	1.394 (2)	C9—H9B	0.97 (2)
C2—H2A	0.96 (2)	C10—H10A	0.97 (2)
C3—C4	1.387 (2)	C10—H10B	0.96 (2)
C3—H3A	1.015 (19)	C10—H10C	0.92 (3)
C4—C5	1.402 (2)	C11—C12	1.516 (2)
C5—C6	1.388 (2)	C11—H11A	0.943 (18)
C5—H5A	1.01 (2)	C11—H11B	1.014 (19)
C6—H6A	0.96 (2)	C12—H12A	0.99 (2)
N2—C9	1.5087 (19)	C12—H12B	0.93 (2)
N2—C7	1.5101 (19)	C12—H12C	0.98 (2)
N2—C11	1.5110 (19)		
			
O3—S1—O5	115.07 (7)	C8—C7—H7A	113.7 (13)
O3—S1—O4	113.04 (7)	N2—C7—H7B	103.5 (12)
O5—S1—O4	111.77 (7)	C8—C7—H7B	113.2 (11)
O3—S1—C4	106.18 (6)	H7A—C7—H7B	109.3 (16)
O5—S1—C4	105.13 (6)	C7—C8—H8A	111.7 (13)
O4—S1—C4	104.57 (6)	C7—C8—H8B	112.2 (11)
O2—N1—O1	123.45 (14)	H8A—C8—H8B	108.7 (16)
O2—N1—C1	118.27 (14)	C7—C8—H8C	109.8 (11)
O1—N1—C1	118.28 (14)	H8A—C8—H8C	113.0 (16)
C2—C1—C6	123.21 (13)	H8B—C8—H8C	101.0 (17)
C2—C1—N1	118.25 (14)	N2—C9—C10	113.09 (13)
C6—C1—N1	118.53 (13)	N2—C9—H9A	106.9 (11)
C1—C2—C3	117.81 (14)	C10—C9—H9A	111.2 (11)
C1—C2—H2A	121.8 (11)	N2—C9—H9B	104.5 (13)
C3—C2—H2A	120.3 (11)	C10—C9—H9B	112.7 (12)
C4—C3—C2	120.31 (13)	H9A—C9—H9B	108.0 (16)
C4—C3—H3A	120.9 (11)	C9—C10—H10A	107.0 (13)
C2—C3—H3A	118.6 (11)	C9—C10—H10B	112.8 (13)
C3—C4—C5	120.74 (13)	H10A—C10—H10B	106 (2)
C3—C4—S1	119.83 (10)	C9—C10—H10C	113.7 (16)
C5—C4—S1	119.42 (11)	H10A—C10—H10C	107.5 (19)
C6—C5—C4	119.75 (14)	H10B—C10—H10C	110 (2)
C6—C5—H5A	120.6 (10)	N2—C11—C12	113.86 (12)
C4—C5—H5A	119.6 (10)	N2—C11—H11A	106.8 (11)
C5—C6—C1	118.18 (14)	C12—C11—H11A	112.1 (11)
C5—C6—H6A	119.3 (14)	N2—C11—H11B	105.6 (10)
C1—C6—H6A	122.6 (14)	C12—C11—H11B	108.3 (11)
C9—N2—C7	110.01 (12)	H11A—C11—H11B	109.9 (15)
C9—N2—C11	113.05 (12)	C11—C12—H12A	113.4 (11)
C7—N2—C11	113.83 (11)	C11—C12—H12B	113.1 (13)
C9—N2—H1N2	110.1 (11)	H12A—C12—H12B	111.1 (17)
C7—N2—H1N2	103.1 (11)	C11—C12—H12C	109.2 (14)
C11—N2—H1N2	106.2 (11)	H12A—C12—H12C	101.6 (17)
N2—C7—C8	113.10 (12)	H12B—C12—H12C	107.7 (18)
N2—C7—H7A	103.1 (12)		
			
O2—N1—C1—C2	−176.81 (16)	O5—S1—C4—C5	−38.73 (14)
O1—N1—C1—C2	2.8 (2)	O4—S1—C4—C5	79.13 (13)
O2—N1—C1—C6	3.1 (2)	C3—C4—C5—C6	−0.4 (2)
O1—N1—C1—C6	−177.33 (18)	S1—C4—C5—C6	−179.65 (12)
C6—C1—C2—C3	−0.9 (2)	C4—C5—C6—C1	0.3 (2)
N1—C1—C2—C3	178.93 (14)	C2—C1—C6—C5	0.4 (3)
C1—C2—C3—C4	0.8 (2)	N1—C1—C6—C5	−179.46 (15)
C2—C3—C4—C5	−0.1 (2)	C9—N2—C7—C8	−179.61 (14)
C2—C3—C4—S1	179.10 (11)	C11—N2—C7—C8	52.33 (18)
O3—S1—C4—C3	19.67 (13)	C7—N2—C9—C10	154.66 (14)
O5—S1—C4—C3	142.05 (12)	C11—N2—C9—C10	−76.86 (17)
O4—S1—C4—C3	−100.09 (12)	C9—N2—C11—C12	−66.52 (16)
O3—S1—C4—C5	−161.10 (12)	C7—N2—C11—C12	59.96 (17)

### Hydrogen-bond geometry (Å, °)

**Table:**

*D*—H···*A*	*D*—H	H···*A*	*D*···*A*	*D*—H···*A*
N2—H1N2···O4	0.95 (2)	1.86 (2)	2.7899 (17)	166 (2)
C2—H2A···O5^i^	0.96 (2)	2.485 (19)	3.1000 (19)	121.7 (14)
C7—H7B···O4^ii^	1.00 (2)	2.50 (2)	3.4081 (19)	151.3 (16)
C10—H10B···O3^iii^	0.96 (2)	2.59 (2)	3.461 (2)	151.1 (18)
C12—H12A···O5^iii^	0.99 (2)	2.60 (2)	3.559 (2)	164.1 (16)

Symmetry codes: (i) −*x*+1/2, *y*−1/2, *z*; (ii) −*x*+3/2, *y*+1/2, *z*; (iii) *x*+1, *y*, *z*.
